# Clinical outcomes of high-risk patients with polycythemia vera after suboptimal response to first-line therapy who switched to ruxolitinib versus nonswitchers: results from the PV-Switch study

**DOI:** 10.1177/20406207251342199

**Published:** 2025-07-04

**Authors:** Steffen Koschmieder, Clemens Schulte, Eyck von der Heyde, Lambert Busque, Françoise Boyer-Perrard, Timothy Devos, Francesco Passamonti, Wendy Y. Cheng, Mu Cheng, Marja Nuortti, Volker Baum, Claire Harrison

**Affiliations:** Department of Hematology, Oncology, Hemostaseology, and Stem Cell Transplantation, Faculty of Medicine, RWTH Aachen University, Pauwelsstr. 30, Aachen 52074, Germany; Center for Integrated Oncology Aachen Bonn Cologne Düsseldorf (CIO ABCD), Aachen, Germany; Gemeinschaftspraxis für Hämatologie und Onkologie, GEFOS, Dortmund, Germany; Medical Oncology, Internal Medicine, Onkologie am Raschplatz, Hannover, Germany; Department of Hematology, Hôpital Maisonneuve-Rosemont, Montreal, QC, Canada; Service des Maladies du sang, Centre Hospitalier Universitaire d’Angers, Angers, France; Department of Hematology, University Hospitals Leuven, Leuven, Belgium; Department of Microbiology and Immunology, Laboratory of Molecular Immunology (Rega Institute), KU Leuven, Leuven, Belgium; Dipartimento di Oncologia ed Onco-Ematologia, Università degli Studi di Milano, Milan, Italy; Fondazione IRCCS Ca’ Granda Ospedale Maggiore Policlinico, Milan, Italy; HEOR, Analysis Group, Inc., Boston, MA, USA; HEOR, Analysis Group, Inc., Boston, MA, USA; Department of Analytics, Global Medical Affairs and Access, Novartis Pharma AG, Basel, Switzerland; Department of Hematology, Novartis Pharma GmbH, Nuernberg, Germany; Department of Haematology, Guy’s and St Thomas’ NHS Foundation Trust, London, UK

**Keywords:** event-free survival, overall survival, polycythemia vera, ruxolitinib, switch

## Abstract

**Background:**

Cytoreductive therapies have been the standard treatment for patients with high-risk polycythemia vera (PV) for decades. However, approximately 24% of patients treated with hydroxyurea will eventually develop resistance or intolerance to hydroxyurea and need second-line (2L) therapy.

**Objective:**

This study compared clinical outcomes of patients with high-risk PV who switched to ruxolitinib as 2L therapy (switchers) versus those who continued first-line (1L) therapy (nonswitchers) after suboptimal response.

**Design:**

This was a retrospective, multicenter, noninterventional study.

**Methods:**

The primary outcome was event-free survival (EFS), defined as the time between the index date and the earliest event of thrombosis, major bleeding, disease progression, or death. Key secondary outcomes included overall survival (OS), time to and rate of disease progression, rate of thrombosis, and change in spleen size.

**Results:**

Overall, 225 patients were included (switchers: 69; nonswitchers: 156). At baseline, >50% of switchers had a prior history of thrombosis (*p* = 0.006) and PV-related symptoms (*p* = 0.037) versus nonswitchers. Switchers had a numerically greater reduction in spleen size at 3 years than nonswitchers (−14.4% vs +15.9%; *p* = 0.107). Compared with nonswitchers, switchers were more likely to experience persistence or presence of new PV-related symptoms as suboptimal response before switching to ruxolitinib (*p* < 0.001). A greater proportion of nonswitchers required ⩾3 phlebotomies to maintain hematocrit <45% within 1 year (*p* < 0.001). No significant differences were observed between switchers and nonswitchers in terms of EFS, OS, time to disease progression, and rate of thrombosis. However, switchers had a significantly higher rate of disease progression to myelofibrosis than nonswitchers (*p* = 0.016).

**Conclusion:**

These data demonstrate the heterogeneity in patient characteristics and type of suboptimal responses between switchers and nonswitchers. The results suggest that patients who switched to ruxolitinib had more severe disease or rapid disease progression and that ruxolitinib may provide some clinical benefit in terms of spleen size reduction and hematocrit control.

## Introduction

Polycythemia vera (PV) is a myeloproliferative neoplasm characterized by trilineage expansion, including an abnormal increase in red cell mass and elevations in platelet and white blood cell (WBC) counts; and it is commonly associated with mutations in the Janus kinase 2 (*JAK2*) gene (V617F or exon 12) leading to hyperactivation of the JAK-STAT signaling pathway.^1,2^ Patients with PV have a considerable symptom burden and are at an increased risk of thrombotic events and progression to myelofibrosis (MF) and blast phase.^3^

Age and prior history of thrombosis have been identified as the most important risk factors for thrombosis in patients with PV; accordingly, patients can be further categorized as high-risk PV (aged >60 years and/or with a history of thrombosis) or low-risk PV (aged ⩽60 years with no history of thrombosis).^4^ Currently, cytoreductive therapy with hydroxyurea and pegylated interferon-α are the most commonly used first-line (1L) treatments for patients with high-risk PV.^4^ However, in clinical practice, 10%–25% of patients with PV who are treated with hydroxyurea develop resistance or intolerance to the drug and require second-line (2L) therapy.^3,5,6^

Several treatment guidelines recommend switching to 2L therapy after suboptimal response to 1L therapy is established in patients with high-risk PV.^5,7,8^ Ruxolitinib, a potent first-in-class *JAK1*/*JAK2* inhibitor, is approved as a 2L therapy for adult patients with PV who are resistant to or intolerant of hydroxyurea and has been shown to provide clinical benefit, including hematocrit and WBC count control and reductions in splenomegaly and symptom burden.^9,10^ Additionally, ruxolitinib has demonstrated reduction in the risk of thrombosis^11–13^ and improvement in total symptom score.^13,14^ Despite the guideline recommendations, many patients continue 1L therapy instead of switching to 2L therapy. Several factors could be associated with the delay in switch, including absence of splenomegaly at baseline, use of low-dose hydroxyurea, lack of access to ruxolitinib in some countries, older age, and presence of certain comorbidities.^15^ The long clinical course of PV makes it difficult to assess long-term clinical outcomes in randomized clinical trials. Therefore, real-world studies are needed to bridge this gap by providing evidence that may help tailor treatment for patients with PV.

This multinational, real-world retrospective chart review study aimed to assess the long-term clinical outcomes, including event-free survival (EFS), overall survival (OS), rate of thrombosis, time to and rate of disease progression, and change in spleen size in patients with high-risk PV who received ruxolitinib as 2L therapy (switchers) versus those who continued receiving 1L therapy (nonswitchers) after suboptimal response.

## Methods

### Study design

**Figure 1.**
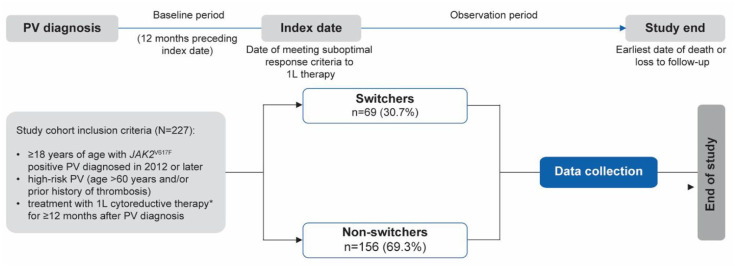
Study design. A minimum of 6 months of follow-up after suboptimal response was mandatory to enter the study, except in the event of death. *1L therapy in this study includes hydroxyurea and pegylated interferon-α. 1L, first-line; PV, polycythemia vera.

This was a multinational, noninterventional, retrospective chart review study with a recruitment target of 350 patients. Data were collected from 18 clinical sites in Belgium, Canada, France, Germany, Italy, the Netherlands, Turkey, and the United Kingdom (Supplemental Table S1). The overall study period (January 30, 2020 to December 31, 2021) included pre- and postindex periods, where the index date was defined as the date of meeting criteria for suboptimal response to 1L cytoreductive therapy. The baseline period was 12 months preceding the index date, and the observation period spanned the time from the index date to the end of data availability or patients’ death (Figure 1).

### Patients

Patients included in the analysis were aged ⩾18 years with *JAK2*^V617F^-positive PV diagnosed in 2012 or later, had high-risk PV (age >60 years and/or prior history of thrombosis), were treated with 1L cytoreductive therapy after PV diagnosis, and met at least one criterion qualifying for suboptimal response to 1L therapy after ⩾12 months of treatment. A minimum of 6 months of follow-up after suboptimal response was mandatory to enter the study, except in the event of death. Patients were excluded if they had initiated a PV treatment other than ruxolitinib after suboptimal response to 1L therapy. Suboptimal response was defined as fulfilling any one of the following criteria: need for ⩾3 phlebotomies within 1 year to maintain hematocrit <45%; leukocyte count >15 × 10^9^/L; platelet count >600 × 10^9^/L; persistence of PV-related symptoms or presence of new PV-related symptoms; cytopenia at the lowest dose of cytoreductive therapy required to achieve a response; and failure to reduce splenomegaly by >50% as measured by palpation, or progressive splenomegaly.

Patients were categorized as either switchers (patients with suboptimal response to 1L therapy who switched to ruxolitinib) or nonswitchers (patients with suboptimal response to 1L therapy who did not switch to ruxolitinib).

### Outcomes

The primary outcome was EFS, defined as the time between the index date and the earliest event of thrombosis, major bleeding, disease progression to MF, accelerated phase (5%–19% blasts in bone marrow), myelodysplastic syndrome (MDS, dysplasia on morphology), or acute myeloid leukemia (AML, ⩾20% blasts in bone marrow), or death. Key secondary outcomes included OS calculated from the index date to death, time to disease progression (determined from the index date to the earliest time of progression to MF, accelerated phase (5%–19% blasts in bone marrow), MDS (dysplasia on morphology), or AML (⩾20% blasts in bone marrow)), rate of disease progression and thrombosis, change in spleen size, molecular profile, and association between potential risk factors and key clinical outcomes in patients diagnosed with PV with suboptimal response.

### Statistical analyses

The target sample size was 350 patients which were justified on the basis of a precision for EFS estimates based on power calculations. Specifically, assuming an equal 1:1 split of the sample size between switchers and nonswitchers, given an assumed EFS rate of 30% for nonswitchers after 36 months of diagnosis, a sample size of 175 nonswitchers would yield a 95% confidence interval (CI) with a width of 14.1% (i.e., with lower limit of 23.3% and upper limit of 37.4%). For switchers, with an assumed event rate of 20%, a sample size of 175 switchers would yield a 95% CI width of 12.4%, with lower limit 14.3% and upper limit 26.7%. As of the cut-off date (December 31st, 2021) for the final analysis, a total of 227 (64.9%) eligible out of the target 350 patients were enrolled in the study. Recruitment was ended early due to substantial delays and hindered capabilities across clinical sites in collecting data, majorly due to coronavirus disease 2019 (COVID-19) pandemic.

Descriptive statistics were used to summarize patient demographics, criteria for suboptimal response, comorbidities (at any time before the index date), PV-related symptoms, and other clinical characteristics during the baseline period. Continuous variables were presented as means and standard deviations, or medians and interquartile ranges; categorical variables were presented as numbers and percentages. The *p* values were estimated using chi-square tests (or Fisher’s exact tests, as appropriate) for categorical variables and using Wilcoxon rank-sum nonparametric tests for continuous variables.

EFS, OS, and time to disease progression were estimated using the Kaplan–Meier (KM) method. Median survival was reported for switchers and nonswitchers with 95% CIs, as well as 25th and 75th percentiles. KM analyses were used to estimate the probabilities of EFS, OS, and rate of events at 12, 24, 36, 48, 60, 72, and 84 months from the index date. Probability of no disease progression was estimated using KM analyses at 12, 24, 36, 48, 60, 72, and 84 months from the index date. The incidence rates of disease progression and thrombosis were calculated by the total number of patients with the event divided by the total person-time of follow-up. The percent change in spleen size (in length or volume) at 1 year (±3 months) and 3 years (±3 months) after the index date was described for both switchers and nonswitchers. The molecular profile (i.e., testing for *JAK2* and extended myeloid mutation testing related to PV) in a limited number of patients was summarized using descriptive statistics for switchers and nonswitchers.

Multivariable analysis was performed using a Cox regression model to identify risk factors for time-to-event endpoints (i.e., EFS, OS, time to disease progression, and thrombosis). Hazard ratios (HRs) and associated 95% CIs were calculated. All analyses were performed using SAS Enterprise Guide software, version 7.1 (SAS Institute, Inc., Cary, NC, USA).

## Results

Overall, 227 patients met the eligibility criteria; two patients experienced progressive disease before the index date and were excluded from the analyses, resulting in a total of 225 patients included in the final analyses were classified as switchers (*n* = 69) or nonswitchers (*n* = 156).

### Patient characteristics

The baseline demographic and clinical characteristics of patients are presented in Table 1. Significant differences in patient clinical characteristics were observed between switchers and nonswitchers at baseline. Switchers were younger than nonswitchers at PV diagnosis (mean age: 64.1 years vs 69.2 years, respectively; *p* = 0.003) and at the index date (66.8 years vs 72.0 years; *p* = 0.002). More than half of the switchers had prior history of thrombosis compared with nonswitchers (52.2% vs 32.7%; *p* = 0.006). A smaller proportion of switchers had received phlebotomies at baseline (34.8% vs 62.2%; *p* < 0.001). The most common 1L cytoreductive therapy was hydroxyurea (97.1% vs 99.4%) followed by pegylated interferon-α (2.9% vs 0.6%) in both switchers and nonswitchers, respectively.

**Table 1.** Demographic^a^ and clinical^b^ characteristics.

Parameter	Switchers (*n* = 69)	Nonswitchers (*n* = 156)	*p* Value^c^
Age at diagnosis, years			
Mean (SD)	64.1 (12.2)	69.2 (9.3)	0.003
Median (IQR)	66.0 (58.0, 72.0)	70.0 (64.0, 75.0)	—
Age at index date, years			
Mean (SD)	66.8 (12.0)	72.0 (9.4)	0.002
Median (IQR)	68.6 (61.4, 74.0)	73.0 (66.5, 78.5)	—
Male, *n* (%)	39 (56.5)	82 (52.6)	0.583
History of thrombosis at the time of diagnosis, *n* (%)	36 (52.2)	51 (32.7)	0.006
Comorbidities before the index date, *n* (%)	46 (66.7)	134 (85.9)	<0.001
Hypertension	29 (42.0)	92 (59.0)	0.019
Cardiovascular	10 (14.5)	34 (21.8)	0.203
Hypercholesterolemia	7 (10.1)	32 (20.5)	0.058
Obesity	7 (10.1)	13 (8.3)	0.660
Cancer	4 (5.8)	19 (12.2)	0.145
Diabetes	2 (2.9)	23 (14.7)	0.009
Other	11 (15.9)	45 (28.8)	0.039
None of the above	23 (33.3)	22 (14.1)	<0.001
PV-related symptoms during the baseline period, *n* (%)	44 (63.8)	76 (48.7)	0.037
Fatigue	19 (27.5)	19 (12.2)	0.005
Pruritus	14 (20.3)	26 (16.7)	0.512
Night sweats	8 (11.6)	3 (1.9)	0.004
Abdominal pain	5 (7.2)	4 (2.6)	0.137
Problems with concentration	2 (2.9)	3 (1.9)	0.644
Inactivity	2 (2.9)	0 (0.0)	0.093
Fever	0 (0.0)	1 (0.6)	1.000
Early satiety	1 (1.4)	3 (1.9)	1.000
Unintentional weight loss	0 (0.0)	3 (1.9)	0.555
Other	6 (8.7)	12 (7.7)	0.798
None of the above	25 (36.2)	80 (51.3)	0.037
Patients who received phlebotomies during the baseline period, *n* (%)	24 (34.8)	97 (62.2)	<0.001
Mean number of phlebotomies (SD)	3.4 (2.1)	3.9 (2.8)	0.551
Median number of phlebotomies (IQR)	2.5 (2.0, 5.0)	3.0 (2.0, 5.0)	—

a On or before the index date.

b During or before the baseline period (i.e., 12 months before the index date).

c *p* Values are estimated using chi-square tests (or Fisher’s exact tests, as appropriate) for categorical variables and Wilcoxon rank-sum nonparametric tests for continuous variables.

IQR, interquartile range; PV, polycythemia vera; SD, standard deviation.

Before the index date, switchers generally had fewer comorbidities than nonswitchers, but switchers were more likely to experience PV-related symptoms (63.8% vs 48.7%; *p* = 0.037); the difference between the two groups was significant for fatigue and night sweats (Table 1).

### Suboptimal response

Switchers had a shorter time from PV diagnosis to suboptimal response than nonswitchers, although this difference was not significant (mean: 32.1 months vs 33.1 months; *p* = 0.194; median: 21.1 months vs 26.0 months), and median time from suboptimal response to switching was 4.6 months (range 0.5–14.5). Distribution of suboptimal response criteria at the index date differed between the two groups (Table 2). Switchers were more likely to experience persistence of PV-related symptoms or presence of new PV-related symptoms as suboptimal response before switching to ruxolitinib compared with nonswitchers (53.6% vs 18.6%; *p* < 0.001). On the contrary, the proportion of nonswitchers who required ⩾3 phlebotomies to maintain hematocrit <45% within 1 year was 36.5% versus 10.1% in switchers (*p* < 0.001). The most common suboptimal response experienced by patients before discontinuing 1L cytoreductive therapy was lack of efficacy (74.2% of patients who switched treatments compared to 23.1% among those who did not switch), followed by intolerable adverse drug reactions (24.2% of patients who switched treatments vs 38.5% among those who did not switch).

**Table 2.** Suboptimal response to first-line cytoreductive therapy at index date.

Parameter	Switchers (*n* = 69)	Nonswitchers (*n* = 156)	*p* Value^a^
Time from diagnosis to suboptimal response, months			
Mean (SD)	32.1 (22.0)	33.1 (19.2)	0.194
Median (IQR)	21.1 (14.8–45.5)	26.0 (18.1–46.5)	—
Time from suboptimal response to switching, months			
Mean (SD)	10.1 (13.9)	—	—
Median (IQR)	4.6 (0.5–14.5)	—	—
Suboptimal response, n (%)			
Need for ⩾3 phlebotomies to maintain hematocrit <45% within 1 year	7 (10.1)	57 (36.5)	<0.001
Leukocyte count >15 × 10^9^/L	11 (15.9)	26 (16.7)	0.892
Platelet count >600 × 10^9^/L	14 (20.3)	33 (21.2)	0.883
Persistence of PV-related symptoms or presence of new PV-related symptoms	37 (53.6)	29 (18.6)	<0.001
Cytopenias at the lowest dose of cytoreductive therapy required to achieve a response	1 (1.4)	8 (5.1)	0.281
Absolute neutrophil count <1.0 × 10^9^/L	0 (0.0)	3 (1.9)	0.555
Platelet count <100 × 10^9^/L	1 (1.4)	3 (1.9)	1.000
Hemoglobin <100 g/L	0 (0.0)	3 (1.9)	0.555
Failure to reduce splenomegaly by >50% as measured by palpation or progressive splenomegaly	5 (7.2)	11 (7.1)	1.000

a *p* Values are estimated using chi-square tests (or Fisher’s exact tests, as appropriate) for categorical variables and Wilcoxon rank-sum nonparametric tests for continuous variables.

IQR, interquartile range; PV, polycythemia vera; SD, standard deviation.

### Spleen assessment

Data for spleen size assessment by palpation were available for 24 switchers (34.8%) and 63 nonswitchers (40.4%) at the index date. The results of spleen assessments at diagnosis or at the index date are presented in Supplemental Table S2. A significantly higher proportion of switchers had massive spleen enlargement than that of nonswitchers at the index date (12.5% vs 0%; *p* = 0.019).

### Event-free survival

**Figure 2.**
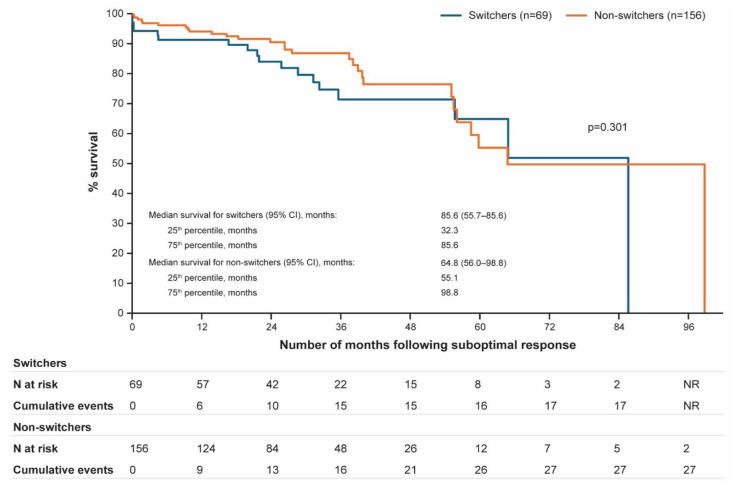
Kaplan–Meier plot for event-free survival. Event-free survival was defined as the time between the index date and the earliest event of thrombosis, major bleeding, disease progression (MF, accelerated phase, MDS or AML), or death. AML, acute myeloid leukemia; CI, confidence interval; MDS, myelodysplastic syndrome; MF, myelofibrosis; *N*, number of patients; NR, not reached.

Median EFS was 85.6 months (95% CI: 55.7–85.6) in switchers and 64.8 months (95% CI: 56.0–98.8) in nonswitchers. At 60 months, the rate of EFS was 64.9% (95% CI: 47.9–72.7) in switchers versus 55.2% (95% CI: 37.8–72.7) in nonswitchers (Figure 2).

### OS and time to disease progression

**Figure 3.**
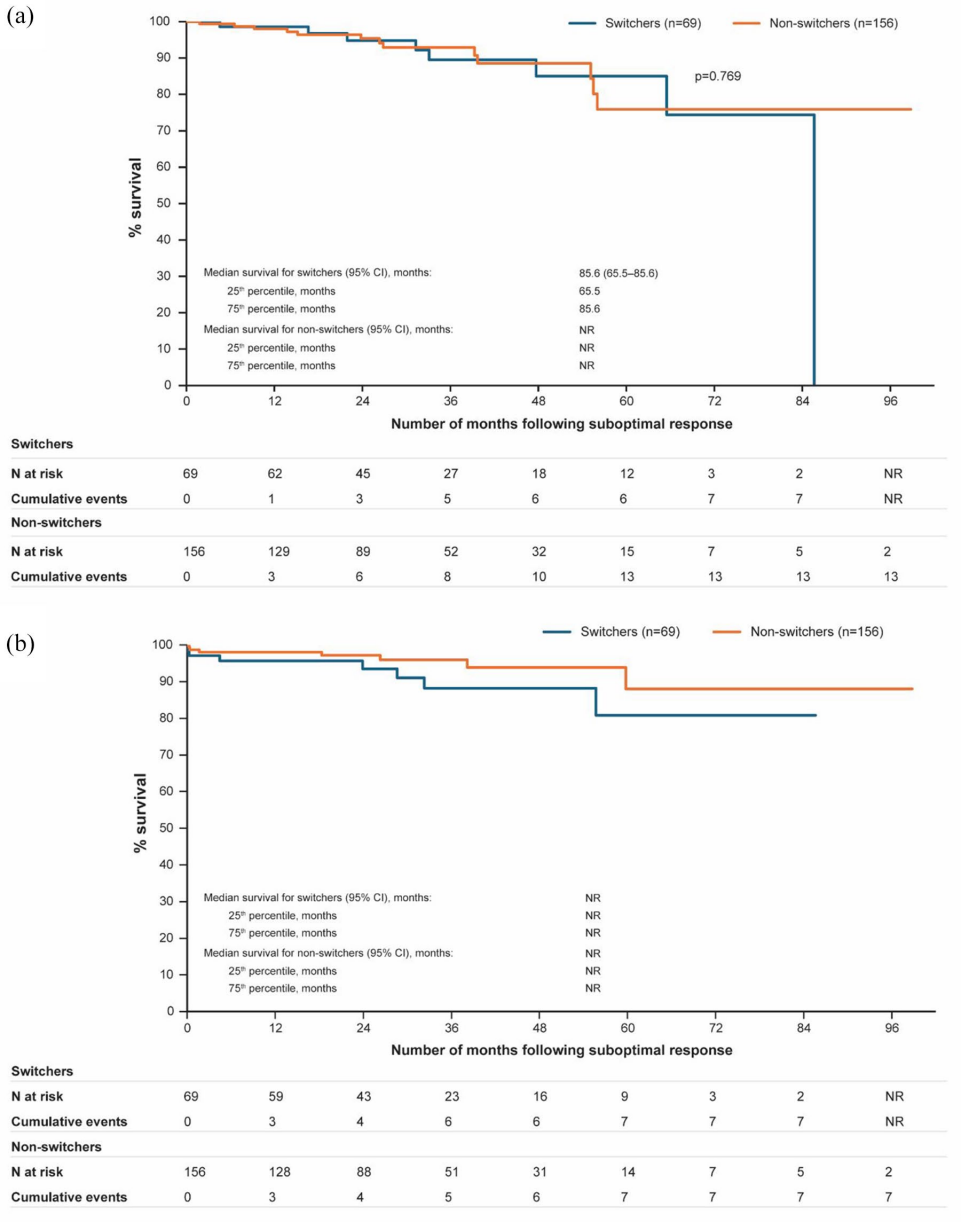
Kaplan–Meier plot for (a) overall survival; (b) time to disease progression among switchers and nonswitchers. CI, confidence interval; N, number of patients; NR, not reached.

No relevant differences were observed between switchers and nonswitchers in terms of survival from 12 to 84 months after suboptimal response was confirmed (Figure 3(a)). Median time to disease progression was not reached in either group due to insufficient number of events (Figure 3(b)). The number of events for both analyses was low.

### Rate of disease progression and thrombosis

Incidence rates of disease progression to MF were significantly higher in switchers than in nonswitchers (34.1 per 1000 person-years (PYs) vs 4.9 per 1000 PYs; incidence rate ratio (IRR): 6.95 (1.44–33.47); *p* = 0.016); however, the overall difference in incidence rates of disease progression between switchers and nonswitchers was not significant. Incidence rates of thrombotic events were 24.4 per 1000 PYs for switchers and 22.1 per 1000 PYs for nonswitchers (*p* = 0.860) during the observation period (Supplemental Table S3).

### Change in spleen size

Among switchers (*n* = 14) and nonswitchers (*n* = 9) with spleen size assessments at 1 year (±3 months) after the index date, the median spleen length decreased by 11.9% in switchers versus an increase of 2.7% in nonswitchers; however, the difference between groups was not significant. This nominal difference in median change in length became larger when the spleen size at 3 years (±3 months) after the index date was compared with that measured at the index date in switchers (−14.4%) and nonswitchers (+15.9%; Supplemental Table S4).

### Molecular profile

The rates of genetic testing were low, particularly for non-*JAK* mutations, in nonswitchers compared with switchers. All patients included in this analysis were *JAK2*-positive. Other than *JAK2*, the most common gene that switchers were tested for was *TET2* (10.1%), followed by *DNMT3A* (8.7%), *ASXL1* (7.2%), *SRSF2* (7.2%), and *IDH2* (7.2%). Among the switchers, three patients (of seven tested, 42.9%) were positive for a *TET2* mutation, and one patient (of six tested,16.7%) was positive for a mutation in the *DNMT3A* gene. In addition to *JAK2*, nonswitchers were tested for mutations in the *BCR::ABL1, CALR*, and *cMPL* (2.6% each) genes; none of the patients tested positive (Supplemental Table S5).

### Risk factors

In multivariable analyses (Table 3), older age at the index date (at 1-year increment) was significantly associated with an increased risk of experiencing an event or death (HR: 1.04; 95% CI: 1.00–1.08; *p* = 0.035) and an increased risk of mortality (HR: 1.09; 95% CI: 1.02–1.16; *p* = 0.009). Males had a fourfold increase in mortality risk compared with females (HR: 4.95; 95% CI: 1.55–15.81; *p* = 0.007). Baseline hematocrit ⩾45% was significantly associated with a lower risk of PV progression than that of <45% (HR: 0.11; 95% CI: 0.01–0.90; *p* = 0.04).

**Table 3.** Multivariable analysis of risk factors for clinical outcomes after the index date.

Variable	EFS		OS		Disease progression	
	HR (95% CI)	*p* Value	HR (95% CI)	*p* Value	HR (95% CI)	*p* Value
Switching to ruxolitinib	1.35 (0.69–2.66)	0.378	1.46 (0.52–4.13)	0.478	1.41 (0.41–4.81)	0.582
Age at index date (years)	1.04 (1.00–1.08)	0.035	1.09 (1.02–1.16)	0.009	1.00 (0.96–1.05)	0.993
Male (Reference: female)	1.57 (0.82–3.01)	0.171	4.95 (1.55–15.81)	0.007	1.08 (0.38–3.04)	0.882
Any baseline comorbidity (Reference: no comorbidity)	1.11 (0.50–2.48)	0.797	2.98 (0.62–14.45)	0.174	0.53 (0.17–1.68)	0.282
Any baseline PV-related symptom (Reference: no PV-related symptom)	0.66 (0.35–1.26)	0.207	0.71 (0.26–1.89)	0.488	0.86 (0.33–2.25)	0.755
Baseline thrombosis or hemorrhage event (Reference: no thrombosis or hemorrhage event)	1.51 (0.80–2.85)	0.203	0.75 (0.28–1.98)	0.559	2.38 (0.86–6.61)	0.097
Phlebotomies received (Reference group: 0)						
1–3 Phlebotomies received	0.58 (0.27–1.23)	0.157	0.32 (0.10–1.00)	0.050	0.94 (0.29–3.03)	0.918
4+ Phlebotomies received	0.56 (0.24–1.30)	0.179	0.31 (0.08–1.19)	0.088	0.48 (0.10–2.35)	0.363
Spleen size (Reference group: normal)						
Splenomegaly	1.00 (0.29–3.42)	0.995	2.06 (0.38–11.28)	0.406	0.70 (0.08–6.28)	0.752
Unknown	1.07 (0.55–2.09)	0.846	1.34 (0.47–3.87)	0.583	0.81 (0.28–2.34)	0.693
Baseline hematocrit level (Reference group: <45%)						
⩾45%	0.76 (0.37–1.57)	0.452	0.90 (0.30–2.67)	0.843	0.11 (0.01–0.90)	0.040
Unknown	0.97 (0.43–2.21)	0.951	1.24 (0.36–4.34)	0.735	0.84 (0.26–2.66)	0.761
Time from diagnosis to suboptimal response (months)	1.01 (0.99–1.03)	0.267	1.01 (0.98–1.04)	0.439	1.02 (0.99–1.04)	0.155

CI, confidence interval; EFS, event-free survival; HR, hazard ratio; OS, overall survival; PV, polycythemia vera.

## Discussion

This real-world, retrospective, noninterventional chart review study provides insights into the demographic and clinical characteristics of patients with PV who had suboptimal response to 1L therapy and switched (switchers) or did not switch (nonswitchers) to ruxolitinib. Despite showing a suboptimal response, most patients (69.3%) remained on 1L cytoreductive therapy instead of switching to 2L therapy with ruxolitinib. Several differences in patient characteristics and type of suboptimal responses were observed between switchers and nonswitchers: switchers had a lower mean age at diagnosis (64.1 years vs 69.2 years) and a shorter time from PV diagnosis to suboptimal response than nonswitchers, whereas more switchers had PV-related symptoms, prior history of thrombosis, and massive splenomegaly than nonswitchers. This observation suggests that patients who switched to ruxolitinib had more severe disease or were experiencing rapid disease progression; the differences in patient characteristics between switchers and nonswitchers also potentially explain the reasons behind treatment change, such as suboptimal symptom management.

In addition to baseline demographic and clinical differences, the types of suboptimal responses experienced by switchers and nonswitchers also differed. Of note, switchers were more likely to experience persistence of PV-related symptoms or presence of new PV-related symptoms as suboptimal response, which was consistent with the reported baseline characteristics, where PV-related symptoms were observed in 64% of switchers. In nonswitchers, the most common type of suboptimal response was related to inadequate hematocrit control, where the need for ⩾3 phlebotomies to maintain hematocrit <45% within 1 year was the most prevalent type of suboptimal response. This is also reflected by the heavy reliance on phlebotomies among nonswitchers (62%) relative to switchers (35%) observed at baseline. These results are consistent with those reported previously, where persistence/occurrence of symptoms were positively associated with treatment switch (hydroxyurea-ruxolitinib vs hydroxyurea-alone/other: 48.1% vs 29.6%, *p* < 0.001), while the need for phlebotomies did not significantly trigger treatment change (58.7% vs 50.2%, *p* = 0.13).^16^

No substantial differences were observed between switchers and nonswitchers for EFS, OS, time to disease progression, and rate of thrombosis. However, the number of patients at the end of the observation period in either group was too small to offer definite evidence for these effectiveness outcomes. In the current study, OS at 60 months was 85.0% for switchers versus 75.9% for nonswitchers, which was slightly lower than that reported in RESPONSE studies (RESPONSE: 91.9% vs 91.0%; RESPONSE-2: 96.0% vs 91.0% for the ruxolitinib and best available therapy (BAT) groups, respectively).^10,17^ Similarly, in a retrospective real-world study of 377 patients with resistance or intolerance to hydroxyurea from the Spanish Registry of Polycythemia Vera, no significant survival benefit was observed with ruxolitinib treatment compared with BAT (HR: 0.8; 95% CI: 0.4–1.7).^18^ Future studies with larger sample sizes and longer follow-up time are needed to validate the findings of this study.

Previous studies reported lower thrombosis rates among patients receiving ruxolitinib than among those receiving BAT.^11,18^ A meta-analysis that utilized data from randomized clinical trials of ruxolitinib demonstrated a consistently lower rate of thrombosis with ruxolitinib than with BAT (IRR: 0.56; 95% CI: 0.28–1.11), corresponding to an incidence of 3.09% and 5.51% per patient-year, respectively, although the difference was not statistically significant.^11^ The real-world study from the Spanish Registry of Polycythemia Vera showed a nonsignificant lower rate of arterial thrombosis among patients treated with ruxolitinib (adjusted IRR: 0.18; 95% CI: 0.02–1.3) and no differences in venous thrombosis rate or major bleeding event rate.^18^ In a real-world study using Electronic Health Record database, patients with PV initially received some protection from thromboembolic events (TE) with hydroxyurea, with the annualized incidence rate reduced to 5.6 (during hydroxyurea) from 8.7 (before hydroxyurea).^19^ However, this effect was not sustained, and an apparent rebound effect was evident over time (incidence rate of 10.5 in patients continuing hydroxyurea), whereas the TE risk remained stable for patients who switched to ruxolitinib. The incidence rate decreased from 10.8 (before hydroxyurea) to 8.4 (during hydroxyurea) and was maintained at 8.3 (after switching to ruxolitinib) in patients who switched to ruxolitinib. In the current study, a nonsignificant difference was observed in thrombotic events between switchers and nonswitchers (24.4 per 1000 PYs for switchers and 22.1 per 1000 PYs for nonswitchers), with an IRR (95% CI) of 1.10 (0.37–3.29). This could be explained by the fact that more than half of the switchers had a prior history of thrombosis at baseline.

Nonswitchers in this study were older and had higher phlebotomy and cardiovascular risk factor burdens than switchers; despite these challenges, patients had relatively favorable outcomes. However, switchers appeared to have better clinical outcomes such as spleen size reduction and hematocrit control relative to nonswitchers. Such differences were observed despite the worse prognosis observed among switchers, which likely reflects more severe disease at baseline in this study population. It is worth mentioning that, in the current study, a nonsignificant reduction in spleen size was observed among switchers at 1 and 3 years after the index date versus an increase in spleen size in nonswitchers for the corresponding time points. This finding is in line with the significant beneficial effect of ruxolitinib in terms of spleen size reduction observed in the RESPONSE and RESPONSE-2 studies.^9,10,17,20^ This study also showed better hematocrit control (<45%) in switchers versus nonswitchers, and a greater proportion of switchers had their laboratory data available (hematocrit <45%) at 1-year postindex date. This finding is also consistent with results from previous ruxolitinib studies.^9,10,17,20^

As this was a retrospective analysis, the study had some limitations. In this study, index date was defined as the time of the first suboptimal response, meaning that reported outcomes (thrombosis, bleeding, and disease progression) may have occurred before switching to ruxolitinib. Moreover, the median time between the first suboptimal response and switching to ruxolitinib was 4.6 months. Furthermore, the observational design of this study restricted frequent monitoring of patients, which generally occurs in clinical trials, leading to incomplete data for spleen size and laboratory outcomes at time points of interest. Identification of patients, disease characteristics (including data on bone marrow fibrosis), treatments, and study outcomes were limited to the availability and accuracy of patient charts and data collection procedures across participating clinical centers. Lastly, patients included in this study were recruited from specific clinical centers, and the sample may not have been representative of the overall population with PV from the participating countries. There may have been inadvertent selection bias related to treatment choice due to differences in drug availability among countries.

## Conclusion

This retrospective chart review study highlights the heterogeneity in patient characteristics and type of suboptimal responses at baseline between patients who switched their treatment to ruxolitinib and nonswitchers. Results from this study showed that clinicians and patients may be more likely to switch to 2L therapy when patients on 1L therapy continue to be symptomatic. No substantial differences in EFS, OS, and rate of thrombosis were observed between switchers and nonswitchers. However, a significantly higher rate of progression to MF was observed in switchers than in nonswitchers. Despite switchers presenting with a more severe disease at baseline (more thrombosis, greater splenomegaly, more symptomatology), the better control of hematocrit and spleen with ruxolitinib potentially allowed switchers to reach an EFS similar to nonswitchers. Future studies with longer follow-up time and larger sample size are warranted to confirm findings from this study.

## Supplemental Material

sj-docx-1-tah-10.1177_20406207251342199 – Supplemental material for Clinical outcomes of high-risk patients with polycythemia vera after suboptimal response to first-line therapy who switched to ruxolitinib versus nonswitchers: results from the PV-Switch study Supplemental material, sj-docx-1-tah-10.1177_20406207251342199 for Clinical outcomes of high-risk patients with polycythemia vera after suboptimal response to first-line therapy who switched to ruxolitinib versus nonswitchers: results from the PV-Switch study by Steffen Koschmieder, Clemens Schulte, Eyck von der Heyde, Lambert Busque, Françoise Boyer-Perrard, Timothy Devos, Francesco Passamonti, Wendy Y. Cheng, Mu Cheng, Marja Nuortti, Volker Baum and Claire Harrison in Therapeutic Advances in Hematology

## Declarations

**Ethics approval and consent to participate:** The study protocol was approved by the authorities and the ethics committees of the clinical centers involved. The study was conducted entirely using retrospective patient charts, and no patient identifiers were requested in this study. Any data, which may reveal patient identity per local regulation, were not collected in electronic case report form. Informed consent was not required in this study as the data collected did not contain personal identifiers. If patient informed consent was deemed as necessary by IRB/IECs and/or local regulations, it was collected prior to any data collection in accordance with sponsor policies and applicable laws and ethical requirements.

**Consent for publication:** IRB approval was obtained for the study; individual consent for publication was not required.

**Availability of data and materials:** Novartis is committed to sharing access to patient-level data and supporting clinical documents from eligible studies with qualified external researchers. These requests are reviewed and approved by an independent review panel based on scientific merit. All data provided are anonymized to respect the privacy of patients who have participated in the trial in line with applicable laws and regulations. This trial data availability is according to the criteria and process described on www.clinicalstudydatarequest.com.
