# Supplementary material for: Clinical outcomes of high-risk patients with polycythemia vera after suboptimal response to first-line therapy who switched to ruxolitinib versus nonswitchers: results from the PV-Switch study
Source: Ther Adv Hematol. 2025 Jul 4;16:20406207251342199. doi: 10.1177/20406207251342199 (PMC12227902; doi:10.1177/20406207251342199)
Supplement: sj-docx-1-tah-10.1177_20406207251342199 – Supplemental material for Clinical outcomes of high-risk patients with polycythemia vera after suboptimal response to first-line therapy who switched to ruxolitinib versus nonswitchers: results from the PV-Switch study [file sj-docx-1-tah-10.1177_20406207251342199.docx]

**Supplemental Table S1. Spleen assessments at the index date**

|  | **Switchers  (n=69)** | **Non-switchers (n=156)** | **p-value**^a^ |
| --- | --- | --- | --- |
| **Spleen length, cm, assessed by imaging, n (%)** | 25 (36.2) | 33 (21.2) |  |
| Mean (SD) | 16.0 (4.9) | 14.5 (3.2) | 0.387 |
| Median (IQR) | 14.6 (12.6–19.3) | 14.1 (13.0–16.0) | - |
| Modality, n (%) |  |  |  |
| Ultrasound | 25 (100.0) | 32 (97.0) | 1.00 |
| Unknown | 0 (0.0) | 1 (3.0) | 1.00 |
| **Spleen size, assessed by palpation, n (%)** | 24 (34.8) | 63 (40.4) | 0.426 |
| Normal | 16 (66.7) | 55 (87.3) | 0.035 |
| Mild splenomegaly | 5 (20.8) | 6 (9.5) | 0.167 |
| Moderate splenomegaly | 0 (0.0) | 2 (3.2) | 1.000 |
| Massive splenomegaly | 3 (12.5) | 0 (0.0) | 0.019 |
| IQR, interquartile range; SD, standard deviation.  ^a^p-values are estimated using chi-square tests (or Fisher’s exact tests, as appropriate) for categorical variables and Wilcoxon rank-sum non-parametric tests for continuous variables. | | | |

**Supplemental Table S2. Rates of disease progression and thrombosis**

|  | **Unadjusted results** | | | | | | | |
| --- | --- | --- | --- | --- | --- | --- | --- | --- |
|  | **Switchers (n=69)** | | | **Non-switchers (n=156)** | | | **IRR (95% CI)** | **p-value** |
|  | **No. of events** | **Person-years of observation** | **IR  (per 1000 person-years)** | **No. of events** | **Person-years of observation** | **IR (per 1000 person-years)** |  |  |
| **Disease progression** | 8 | 205.0 | 39.0 | 10 | 407.3 | 24.6 | 1.59 (0.63–4.03) | 0.329 |
| MF | 7 | 205.0 | 34.1 | 2 | 407.3 | 4.9 | 6.95 (1.44–33.47) | 0.016 |
| Accelerated phase^a^ | 0 | 205.0 | 0.0 | 2 | 407.3 | 4.9 | - | - |
| MDS^b^ | 1 | 205.0 | 4.9 | 1 | 407.3 | 2.5 | 1.99 (0.12–31.76) | 0.627 |
| AML^c^ | 0 | 205.0 | 0.0 | 5 | 407.3 | 12.3 | - | - |
| **Thrombotic events** | 5 | 205.0 | 24.4 | 9 | 407.3 | 22.1 | 1.10 (0.37–3.29) | 0.860 |
| Arterial thrombosis | 1 | 205.0 | 4.9 | 7 | 407.3 | 17.2 | 0.28 (0.03–2.31) | 0.239 |
| Venous thrombosis | 4 | 205.0 | 19.5 | 2 | 407.3 | 4.9 | 3.97 (0.73–21.69) | 0.111 |
| Blue toe syndrome | 0 | 205.0 | 0.0 | 0 | 407.3 | 0.0 | - | - |
| Extremity necrosis | 0 | 205.0 | 0.0 | 0 | 407.3 | 0.0 | - | - |
| AML, acute myeloid leukemia; CI, confidence interval; IR, incidence rate; IRR, incidence rate ratio; MDS, myelodysplastic syndrome; MF, myelofibrosis.  ^a^Accelerated phase is defined by 5%–19% of blasts in the bone marrow.  ^b^MDS is defined by dysplasia on morphology.  ^c^AML is defined by ≥20% of blasts in the bone marrow. | | | | | | | | |

**Supplemental Table S3. Change in spleen size after the index date**

| **Spleen size** | **Switchers  (n=69)** | **Non-switchers (n=156)** | **p-value**^b^ |
| --- | --- | --- | --- |
| **At 1 year (±3 months) after the index date, n (%)** | 20 (29.0) | 20 (12.8) |  |
| Spleen size reduction evaluable^a^, n (%) | 14 (20.3) | 9 (5.8) |  |
| Change in length, % |  |  |  |
| Mean (SD) | −3.3 (25.3) | 4.1 (9.1) | 0.115 |
| Median (IQR) | −11.9 (−18.1, 10.0) | 2.7 (−0.6, 7.7) | - |
| **At 3 years (±3 months) after the index date, n (%)** | 11 (15.9) | 9 (5.8) |  |
| Spleen size reduction evaluable^a^, n (%) | 8 (11.6) | 4 (2.6) |  |
| Change in length, % |  |  |  |
| Mean (SD) | −7.5 (22.1) | 19.4 (15.9) | 0.107 |
| Median (IQR) | −14.4 (−24.9, 11.4) | 15.9 (9.6, 29.1) | - |
| IQR, interquartile range; SD, standard deviation.  ^a^Spleen size reduction was evaluated in patients who had spleen length assessed both on the index date and at a specific time point.  ^b^p-values are estimated using chi-square tests (or Fisher’s exact tests, as appropriate) for categorical variables and Wilcoxon rank-sum non-parametric tests for continuous variables. | | | |

**Supplemental Table S4. Molecular profile**

|  | **Switchers**  **(n=69)** | **Non-switchers (n=156)** | **p-value^a^** |
| --- | --- | --- | --- |
| **Genetic test performed, n (%)** |  |  |  |
| ***JAK2*^V617F^** | 17 (24.6) | 12 (7.7) | <0.001 |
| Positive | 17 (100.0) | 11 (91.7) | 0.414 |
| Negative | 0 (0.0) | 1 (8.3) |  |
| ***ASXL1*** | 5 (7.2) | 0 (0.0) | 0.002 |
| Positive | 0 (0.0) | 0 (0.0) | N/A |
| Negative | 5 (100.0) | 0 (0.0) |  |
| ***RUNX1*** | 1 (1.4) | 0 (0.0) | 0.307 |
| Positive | 0 (0.0) | 0 (0.0) | N/A |
| Negative | 1 (100.0) | 0 (0.0) |  |
| ***SRSF2*** | 5 (7.2) | 0 (0.0) | 0.002 |
| Positive | 0 (0.0) | 0 (0.0) | N/A |
| Negative | 5 (100.0) | 0 (0.0) |  |
| ***DNMT3A*** | 6 (8.7) | 0 (0.0) | <0.001 |
| Positive | 1 (16.7) | 0 (0.0) | N/A |
| Negative | 5 (83.3) | 0 (0.0) |  |
| ***TET2*** | 7 (10.1) | 0 (0.0) | <0.001 |
| Positive | 3 (42.9) | 0 (0.0) | N/A |
| Negative | 4 (57.1) | 0 (0.0) |  |
| ***IDH2*** | 5 (7.2) | 0 (0.0) | 0.002 |
| Positive | 0 (0.0) | 0 (0.0) | N/A |
| Negative | 5 (100.0) | 0 (0.0) |  |
| **Other^b^** | 6 (8.7) | 9 (5.8) | 0.401 |
| **None** | 47 (68.1) | 141 (90.4) | <0.001 |
| qPCR, quantitative polymerase chain reaction.  ^a^p-values are estimated using chi-square tests (or Fisher’s exact tests, as appropriate) for categorical variables and Wilcoxon rank-sum non-parametric tests for continuous variables.  ^b^Other genetic tests performed included the following:   - Switchers: *EZH2* (n=3, positive=0), *SMC3* (n=1, positive=1), *CALR* and *cMPL* (n=2, positive=0). - Non-switchers: *BCR::ABL1* (n=4, positive=0), *PRV1* qPCR (n=1, n positive=1), *JAK2* exon12 (n=1, positive=1), and *CALR* and *cMPL* (n=4, positive=0). | | | |

**Supplemental Table S5. List of Independent Ethics Committees (IEC) or Institutional Review Boards (IRB) by study center**

| Center No. | Ethics Committee or Institutional Review Board | City | Country |
| --- | --- | --- | --- |
| 1 | Ethics Committee Research  UZ/KU Leuven | Leuven | Belgium |
| 2 | Commissie voor Medische Ethiek ZNA, Institutional Review Board - ZNA/OCMW | Antwerpen | Belgium |
| 3 | CIUSSS de l'Est-de-l'île-de-Montréal | Québec | Canada |
| 4 | UHN Research Ethics Board | Toronto | Canada |
| 5 | Commission nationale informatique et libertés | Paris | France |
| 6 | Ethik-Kommission an der medizinischen Fakultät der RWTH Aachen | Aachen | Germany |
| 7 | Ethik-Kommission bei der Landesärztekammer Rheinland-Pfalz | Mainz | Germany |
| 8 | Ethik-Kommission bei der Landesärztekammer Rheinland-Pfalz | Mainz | Germany |
| 9 | Comitato Etico Fondazione Policlinico Universitario Agostino Gemelli IRCCS – Università Cattolica del Sacro Cuore | Roma | Italy |
| 10 | Comitato Etico di Area Vasta Emilia Centro della Regione Emilia-Romagna (CE-AVEC) | Bologna | Italy |
| 11 | Azienda Ospedaliero-Universitaria Policlinico "G. Rodolico- San Marco"" Catania Comitato Etico Catania 1 | Catania | Italy |
| 12 | Ethics Committee of Insubria | Varese | Italy |
| 13 | Comité de ética de la investigación Hospital Universitario Ramón y Cajal | Madrid | Spain |
| 14 | Marmara üniversitesi klinik araştırmalar etik kurulu | İstanbul | Turkey |
| 15 | Marmara üniversitesi klinik araştırmalar etik kurulu | İstanbul | Turkey |
| 16 | Marmara üniversitesi klinik araştırmalar etik kurulu | İstanbul | Turkey |
| 17 | NHS Health Research Authority | London | UK |
